# A Review of Probiotic Supplementation and Its Impact on the Health and Well-Being of Domestic Cats

**DOI:** 10.3390/vetsci12080703

**Published:** 2025-07-28

**Authors:** Bhagavathi Sundaram Sivamaruthi, Periyanaina Kesika, Chaiyavat Chaiyasut, Pranom Fukngoen, Natarajan Sisubalan

**Affiliations:** 1 Innovation Center for Holistic Health, Nutraceuticals, and Cosmeceuticals, Faculty of Pharmacy, Chiang Mai University, Chiang Mai 50200, Thailand; sivamaruthi.b@cmu.ac.th (B.S.S.); kesika.p@cmu.ac.th (P.K.); chaiyavat@gmail.com (C.C.); 2 Office of Research Administration, Chiang Mai University, Chiang Mai 50200, Thailand; 3 Department of Microbiology, Karpagam Academy of Higher Education, Coimbatore 641021, India; 4 PG and Research Department of Botany, Pachaiyappa’s College, Chennai 600030, India

**Keywords:** probiotics, cats, microbiota, inflammation, immune response, *Lactobacillus*, *Bifidobacterium*

## Abstract

Probiotic supplementation in domestic cats has emerged as a promising non-pharmaceutical strategy to enhance gut health, immune function, and overall well-being. This review critically examines the current literature on probiotic use in feline health, highlighting evidence from studies involving both healthy and diseased cats. Probiotic strains such as *Lactobacillus*, *Bifidobacterium*, *Bacillus*, *Enterococcus*, and *Saccharomyces* have demonstrated beneficial effects, including the modulation of the gut microbiota, a reduction in inflammation, and an improvement in gastrointestinal symptoms. Mechanistically, probiotics exert effects through microbial competition, the enhancement of epithelial barrier function, and immune modulation via cytokine and antimicrobial peptide regulation. Despite promising outcomes, limitations such as short study durations, small sample sizes, and narrow breed diversity constrain generalizability. Future research should prioritize long-term, multi-omics-integrated studies to elucidate mechanisms and optimize clinical application. Overall, probiotics offer a safe, functional dietary tool for improving feline health and may complement conventional veterinary care.

## Simple Summary

Probiotics are gaining attention as a safe, functional dietary intervention to improve the health of domestic cats, particularly by enhancing gut microbiota, strengthening the immune system, and reducing inflammation. Studies have shown that various probiotic strains can benefit both healthy and sick cats; however, more long-term, large-scale research is needed. Overall, probiotics hold strong potential for integration into routine feline healthcare.

## 1. Introduction

In recent years, pets have become increasingly important members of households, leading owners to prioritize their animals’ quality of life [1]. This shift in attitude is mirrored by the rapid expansion of the global pet product industry, which was valued at USD 184.4 billion in 2023 [2]. Despite the growing number of kittens, one of the primary health challenges they face is the underdevelopment of their immune system, which renders them more susceptible to infections and various illnesses, ultimately affecting their overall health and survival [3,4].

As a result of these challenges, pet owners have shown an increased interest in the welfare of their animals, particularly regarding the composition and production standards of commercial pet food [5]. While cats are obligate carnivores and naturally thrive on high-protein diets, lifestyle changes, especially in urban environments, have led to a higher intake of carbohydrates, introducing new health concerns [6]. Like humans, companion animals rely heavily on a balanced gut microbiota for maintaining overall health [7].

This is especially important during the early life stages, as kittens possess an immature immune system and rely on maternal antibodies obtained through colostrum for protection. The weaning period disrupts this passive immunity, resulting in reduced antibody levels and increased susceptibility to infections [8]. Immunocompromised kittens often exhibit signs such as delayed growth, poor coat quality, lethargy, and gastrointestinal issues, including diarrhea and vomiting [9,10,11,12]. These symptoms are often associated with a fragile intestinal barrier and an impaired ability to fight off pathogens. Although antibiotics are commonly used in such cases, their overuse can lead to antimicrobial resistance and negatively impact the gut microbiome, which plays a crucial role in immune function [13].

The use of antibiotics in cats may lead to significant issues such as antimicrobial resistance, gut microbiota disruption, and drug toxicity due to species-specific metabolic limitations [14]. Adverse effects may include gastrointestinal disturbances, immune dysregulation, and even retinal degeneration with certain drugs like enrofloxacin [15]. To counter these challenges, nutritional interventions such as lactoferrin and probiotics have emerged as promising alternatives to antibiotics, offering support to the immune system without disrupting microbial balance [16,17]. Probiotics have garnered attention for their potential to improve pet health. They are defined as “live microorganisms that, when administered in adequate amounts, confer a health benefit on the host” [18]. These beneficial microbes, originating from sources such as commensal gut bacteria and fermented foods, must be well characterized, proven effective in controlled studies, and verified for safety [19].

In pets, probiotics have been linked to multiple benefits [19], including enhanced immune response, reduced stress, defense against intestinal pathogens, and improved growth and development. This growing interest aligns with pet owners’ increasing desire to prolong their animals’ lifespan and quality of life [20].

Among probiotics, *Bacillus* spp. are known for producing bacteriocins and other antimicrobial compounds. These substances offer various advantages, including immunomodulation, vitamin synthesis, and the protection of intestinal tissues against harmful agents [21]. Although several studies have examined bacteriocins from various *Bacillus* strains [22,23], there is limited research specifically evaluating *B. clausii* supplementation in cats. However, in human studies, *B. clausii* has demonstrated positive clinical outcomes, particularly in managing diarrhea and preventing infections [24].

The gut microbiota plays a vital role in host metabolism, influencing energy regulation, glucose utilization, and lipid metabolism [25]. This microbial community supports a range of functions, including gastrointestinal development, maintaining barrier integrity, modulating the immune system, resisting pathogens, and producing essential nutrients. Its impact extends beyond the gut, affecting systemic health as well [26,27,28,29,30,31,32,33]. Among the short-chain fatty acids (SCFAs) produced by gut microbes, butyrate is particularly significant as an energy source for colonocytes, offering several intestinal and extraintestinal benefits, including anti-inflammatory and absorptive effects [34].

Probiotic strains from the *Lactobacillus* genus have shown promise in enhancing both gut and immune health [17]. For instance, Lubbs et al. [35] found that supplementing cats with *L. acidophilus* altered fecal microbiota and increased the presence of beneficial bacteria [35]. These findings suggest that while mechanisms may differ from those in omnivores, probiotics can still significantly enhance feline gut health. Moreover, additional research suggests that *Lactobacillus* strains can enhance protein levels, strengthen immune responses, improve gastrointestinal comfort, and influence various metabolic and inflammatory markers [36,37].

This review assesses the effects of probiotic supplementation on feline health, with an emphasis on underlying mechanisms. It outlines the role of the gut microbiota in overall health, examines how probiotics modulate microbial balance, and explores their immunomodulatory and therapeutic potential.

## 2. Microbiota of Felines

The gut microbiota plays a vital role in maintaining the overall health and metabolic balance of domestic cats. Understanding its composition, diversity, and responsiveness to dietary or probiotic interventions is fundamental to evaluating the health impacts of supplementation strategies.

Multiple factors influence the composition of the feline gut microbiota, including age, sex, diet, breed, general health status, and even geographic location. Firmicutes are often the dominant phylum in healthy cats, with lower proportions of Actinobacteria and Bacteroidetes [38]. In general, the feline gut microbiota is predominantly composed of four bacterial phyla: Firmicutes, Actinobacteria, Bacteroidetes, and Actinobacteria [39].

Pathological conditions also correlate with notable microbial changes. In diarrheal cats, an observed increase was noted in Burkholderiales, Enterobacteriaceae, *Streptococcus*, and *Collinsella*, whereas healthy cats exhibited higher levels of Campylobacterales, Bacteroidaceae, *Megamonas*, *Helicobacter*, and *Roseburia*. Moreover, cats with acute diarrhea showed reductions in Erysipelotrichia and *Lactobacillus*, while chronic cases demonstrated a decrease in Bacteroidetes compared with healthy controls [40]. Inflammatory conditions, such as feline inflammatory bowel disease (IBD), were associated with decreased Firmicutes and Bacteroidetes, increased Proteobacteria, and reduced diversity in beneficial groups, including *Clostridium* clusters XIVa and IV [28]. Similarly, cats suffering from chronic enteropathies, including IBD and small-cell lymphoma, exhibited significantly reduced alpha diversity, indicating compromised microbial richness and resilience [41].

A total of 20 healthy kittens, all aged two months and evenly divided by sex, were selected for the study. These included British shorthair blue cats (*n* = 4), British shorthair gradient cats (*n* = 4), Siamese cats (*n* = 6), and American shorthair cats (*n* = 6), with each breed comprising individuals from two separate litters. The kittens had a consistent body weight, with an average of 0.90 ± 0.10 kg. At the genus level, an analysis of the gut microbiota revealed the top ten most abundant bacterial genera in each group: *Peptoclostridium*, *Collinsella*, *Olsenella*, *Blautia*, *Prevotella*, *Megasphaera*, *Bacteroides*, *Bifidobacterium*, *Dialister*, and *Aloprevotella*. A significant increase in the abundance of *Ruminococcus*, *Libanicoccus*, and *Marvinbryanlia* was observed by day 4 after weaning, compared with day 0. By day 30 post weaning, the levels of *Bifidobacterium*, *Holdemanella*, and *Solobacterium* had significantly increased [42].

Conversely, genera such as *Bacteroides*, *Parabacteroides*, *Fusobacterium*, and *Odoribacter* showed a marked decline on day 4 post weaning when compared with their abundance on day 30 post weaning. Furthermore, there was a notable upward trend in the abundance of the phylum Actinobacteria. Within this phylum, *Bifidobacterium* emerged as the dominant genus, suggesting a potentially vital role in maintaining the health of kittens following weaning [42]. Alongside *Bifidobacterium*, *Prevotella* also showed an increase in abundance after weaning. These genera belong to the Actinobacteria phylum, which is known to participate in the fermentation of plant-derived non-starch polysaccharides into short-chain fatty acids [43,44].

Overall, five bacterial genera, *Lactobacillus*, *Bifidobacterium*, *Bacteroides*, *Prevotella*, and *Megasphaera,* were identified as key contributors to the structural shifts observed in the fecal microbiome during the weaning period [45]. Among the *Bacteroides* species, declines were recorded for *Bacteroides fragilis*, *Bacteroides vulgatus*, and *Bacteroides stercoris*. Prior research indicates that *Bacteroides fragilis* plays a protective role against *Clostridium difficile* infection, potentially by facilitating probiotic colonization, impeding pathogen establishment, and enhancing the integrity of the intestinal barrier [46].

These findings collectively highlight the dynamic nature of the feline microbiota and its sensitivity to both health status and dietary interventions. A deeper understanding of these microbial communities and their functional roles is crucial for informing the development of targeted probiotic therapies. Moving forward, integrating microbiome characterization into clinical studies will be crucial for uncovering the mechanisms by which probiotics modulate feline health and disease. Very limited studies have detailed the microbiota of cats; further studies are required to reveal the normal flora and their changes during diseased conditions.

## 3. Measurements of the Well-Being of Cats

Evaluating the well-being of cats requires a multi-faceted approach, including blood markers, hormonal profiling, and behavioral or activity-based assessments. These parameters measure the health, stress, and physiological stability of cats.

Structured behavioral assessments, such as ordinal rating scales (used to assess anxiety, fear, petting response, play behavior, and feed intake), trait rating, and qualitative behavior histories, are widely used to monitor cat welfare [47]. The hormonal biomarker (cortisol) has been used to measure chronic stress [48]. The quality of life for cats can be measured using 32 unique tools, as detailed in a recent literature review [49].

Variations in erythrocytes, leukocytes, and platelets indicate changes in immune status or the presence of pathology. The neutrophil-to-lymphocyte (N:L) ratio serves as a proxy for chronic stress. A high N:L ratio correlates with sustained cortisol elevation. Specifically, serum or fecal cortisol levels reflect acute or chronic stress responses. The hematological parameters, such as alanine aminotransferase (ALT), creatinine, urea, and amylase, are used to assess organ function and metabolic health. Pavlova et al. [50] reported the physiological health of wild felids (Far Eastern leopards, Pallas cats, and Siberian tigers) as a proxy for their welfare, using fecal cortisol levels, hematological profiles, and serological screening for infectious diseases. Among the species, Pallas cats exhibited the lowest N:L ratio and pathogen exposure, indicating minimal stress and robust immune function. In contrast, leopards had the highest N:L ratios and were positive for multiple pathogens, suggesting higher stress and immunosuppression, which may have been exacerbated by prolonged capture and handling. Collectively, these indicators provide a comprehensive toolset for evaluating the welfare of wild felids and informing conservation efforts [51]. It has been reported that handling stress significantly altered WBC counts and cortisol levels in *Prionailurus bengalensis* cats. Additionally, the study found that the N:L ratio was not affected when samples were collected within 1 h and analyzed [50].

The standard reference values for key blood parameters in Van cats, a culturally important and protected breed native to Turkey, have been attempted. Blood samples from healthy individuals were analyzed to assess a wide spectrum of biochemical markers, including glucose (44–143 mg/dL), urea (11.1–82 mg/dL), creatinine (0.4–3.3 mg/dL), total protein (1.63–14.0 g/dL), bilirubin (0.1–1.0 mg/dL), lipids (54–989 mg/dL), cholesterol (42.6–120 mg/dL), and several enzymes associated with the liver and muscle [such as amylase (270–1552 U/L), ALT (8–72 U/L), aspartate aminotransferase (9–59 U/L), alkaline phosphatase (11.5–82 U/L), creatine kinase (18–161 U/L), lactate dehydrogenase (96–258 U/L), sorbitol dehydrogenase (1.6–12.8 U/L), cholinesterase (474–3060 U/L), creatine kinase (18–161 U/L), alpha-hydroxybutyrate dehydrogenase (49–164 U/L), and glutamyl transpeptidase (3.0–8.4 U/L)]. Additionally, serum protein electrophoresis has been employed to measure specific protein fractions, including albumin (2.53–8.47 g/dL) and α1- (0.11–0.35 g/dL), α2- (0.88–1.76 g/dL), β- (0.48–1.4 g/dL), and γ-globulins (0.79–2.79 g/dL). The observed minimum and maximum values of the parameters are detailed. While most values were consistent with those found in general feline populations, some enzymatic activities appeared unique to Van cats. The study highlighted the significance of these results in providing a reliable reference for veterinary diagnostics and enhancing the health monitoring and preservation of this distinctive breed [52].

Behavioral assessment is a critical component in evaluating the welfare of cats, particularly in shelters, veterinary clinics, and domestic settings. Validated tools, such as ordinal rating scales, have been developed to quantify behavioral indicators like fear, anxiety, playfulness, and food intake on a scale of zero to five, demonstrating excellent interobserver reliability when supported by proper training protocols [47]. Play behavior, in particular, has been shown to correlate strongly with feline well-being. A global study involving over 1500 cat guardians found that frequent and diverse play activities were associated with a higher quality of life and stronger human–cat relationships. In contrast, the absence of play was correlated with signs of emotional distress [53]. In shelter environments, the cat stress score remains the most widely used behavioral tool. However, its correlation with physiological stress markers is inconsistent, suggesting that behavioral observations should be integrated with physical assessments for a comprehensive view of welfare [54]. Observations of cats at public exhibitions also show that well-socialized cats may tolerate stressful stimuli, although behaviors such as hiding increase during peak visitor times, indicating context-dependent stress responses [55]. Despite these advancements, many cat owners struggle to recognize subtle stress behaviors, such as overgrooming or social withdrawal, which can lead to an underestimation of their pets’ stress levels [56]. Overall, precise behavioral assessment in cats depends on several factors, including the use of validated tools and observer training.

The health status of cats was also closely associated with their gastrointestinal health. It plays a key role in overall well-being, influencing digestion, nutrient absorption, immune responses, and mental health. It has been reported that there is a significant difference in the gut microbiota of healthy cats and those with inflammatory bowel disease or alimentary small cell lymphoma, highlighting the possible link between gut microbial composition and the incidence of disease and its development [41]. A healthy microbiota helps strengthen the host’s antioxidant and immune systems [57]. Bugrov et al. [58] reported that reduced microbial diversity could weaken the immunity of cats [58]. Healthy feline microbiota is dominated by the phyla Firmicutes, Bacteroidetes, Actinobacteria, and Proteobacteria, and the genera *Clostridium, Lactobacillus*, *Bacteroides*, *Prevotella*, and *Escherichia*. Diet, age, the use of antibiotics, and underlying health conditions are the most influential factors of healthy microbiota. High-protein, low-carb diets support *Clostridium* and *Fusobacterium*. Fiber inclusion increases *Bacteroides*, *Prevotella*, and short-chain fatty acid producers. Raw meat diets boost proteolytic bacteria, while commercial dry diets may reduce diversity. Regarding age, kittens show lower microbial richness, and the diversity increases with age, stabilizing in adulthood. Aged cats may experience a decline in diversity, similar to humans.

The use of broad-spectrum antibiotics (like metronidazole) dramatically reduces microbial diversity. The recovery of healthy microbiota may take weeks to months and may lead to dysbiosis or an increased susceptibility to pathogens.

Health conditions like inflammatory bowel disease (decreased Firmicutes and Bacteroides, increased Enterobacteriaceae), chronic diarrhea (associated with a low diversity and enrichment of *C. perfringens*), obesity (Firmicutes dominance and a reduction in Bacteroidetes), and diabetes affect the healthy microbiota [58].

## 4. Health Benefits of Probiotic Supplementation in Domestic Cats

Probiotic supplementation in domestic cats has gained attention for its potential to enhance gut health, modulate immune responses, and support overall well-being. Various studies have explored these benefits in healthy feline populations, revealing improvements in microbiota composition, fecal quality, and metabolic function (Table 1).

Probiotic and synbiotic supplementation have been widely studied for their ability to beneficially modulate the gut microbiota and support systemic health in domestic cats. Marshall-Jones et al. [59] investigated the impact of supplementing healthy adult cats with the probiotic *L. acidophilus* DSM13241. Fifteen cats were supplemented with a dry diet and *L. acidophilus* DSM13241 (2 × 10^8^ CFU/day) for 4.5 weeks. After the study period, the changes in fecal microbiota were assessed. The results indicated that probiotic supplementation resulted in an increased abundance of beneficial gut bacteria, particularly *Lactobacillus* spp., while reducing the levels of potentially harmful bacteria such as *Clostridium* spp. and *E. faecalis*. Additionally, a decrease in fecal pH was observed, indicating a gut environment more favorable for lactic acid bacteria (LAB) proliferation. Additionally, systemic benefits were observed, including changes in white blood cell profiles, enhanced granulocyte phagocytic activity, decreased plasma endotoxin levels, and reduced red blood cell susceptibility to osmotic stress. These findings support the role of *L. acidophilus* DSM13241 in positively influencing gut microbiota and enhancing immune function in cats [59].

Garcia-Mazcorro et al. [60] evaluated the impact of a multi-strain synbiotic formulation, Proviable^®^-DC, on the fecal microbiota of healthy cats. The synbiotic, containing seven probiotic strains (5 × 10^9^ CFU) along with prebiotic components such as fructo-oligosaccharides and arabinogalactans, was administered daily for 21 days. Fecal and serum samples were collected before, during, and after the intervention. Probiotic strains were detected in the feces of most animals during the supplementation period, with a notable increase in *Enterococcus* and *Streptococcus* spp., which normalized after discontinuation. Despite these shifts, the overall composition of the dominant bacterial phyla remained unchanged as assessed by advanced molecular techniques, including 16S rRNA gene sequencing and real-time PCR. Importantly, no adverse gastrointestinal effects or significant changes in immune parameters were noted. The findings suggest that while this synbiotic enhances the presence of beneficial bacteria in feces, it does not significantly alter the core microbiota in healthy animals [60]. In another study, a synbiotic combination (*Bif. pseudocatenulatum* strain BP-B82 and galacto-oligosaccharides; GOS) was evaluated for its effects on the gut microbiota of healthy adult cats. The probiotic strain, originally isolated from feline sources, was assessed for its growth performance on various substrates, and GOS was identified as the optimal prebiotic for pairing. The cats were supplemented with a daily dose of 1% GOS-BP-B82, delivering 10^10^ CFU per day for 15 days. Fecal analysis showed a significant reduction in ammonia levels and an increase in acetic acid concentration during the supplementation phase. Additionally, lactic, n-valeric, and isovaleric acid concentrations decreased significantly mid-intervention, before partially returning to baseline. Notably, fecal bifidobacterial counts increased markedly by day 16 and remained elevated through day 25 compared with baseline values. These results suggest that this specific synbiotic combination may support beneficial modifications to the feline intestinal microbiota [61].

Furthermore, a randomized, double-blind, placebo-controlled crossover study assessed the effectiveness of synbiotic supplementation in reducing antibiotic-associated gastrointestinal signs (AAGSs) in healthy cats treated with clindamycin. During two study periods separated by a 6-week washout, cats received clindamycin (75 mg once daily) followed by either a synbiotic or placebo. Key clinical parameters, including food intake, vomiting, fecal consistency, and treatment completion, were monitored. The cats given the synbiotic were significantly more likely to complete the full course of clindamycin during the first treatment period and exhibited a higher food intake throughout the treatment. While vomiting was reduced in the synbiotic group, the difference was not statistically significant. Interestingly, synbiotic administration did not significantly affect fecal scores, which generally worsened over time regardless of treatment. The study also noted lasting clinical benefits from the synbiotic, with a reduction in chronic gastrointestinal signs observed even six weeks post treatment. These findings suggest that synbiotics may help reduce certain AAGSs, especially hyporexia and vomiting, thereby improving treatment compliance and potentially reducing the risk of antimicrobial resistance by supporting full antibiotic course completion. Further research into clinically ill animals is recommended to validate these outcomes [62].

Likewise, a study conducted by Fusi et al. [63] assessed the impact of the probiotic strain *L. acidophilus* D2/CSL (CECT 4529) on fecal quality and overall nutritional status in healthy adult cats. Ten cats were randomly divided into two groups: a control group receiving a standard commercial dry diet, and a treatment group receiving the same diet supplemented with the probiotic at a minimum concentration of 5 × 10^9^ CFU/kg of feed. Over five weeks, body weight and body condition score were monitored to evaluate nutritional status, while fecal score, fecal moisture, and bacterial counts were used to assess gastrointestinal health. No significant differences were observed in body weight or body condition between the groups. However, the cats in the probiotic group exhibited improved fecal quality, with lower fecal moisture and a fecal score closer to the ideal range. A microbiological analysis revealed an increase in fecal *Lactobacillus* counts and a decrease in *Es. coli* levels in the treated group. These preliminary results suggest that the dietary inclusion of *L. acidophilus* D2/CSL may improve fecal consistency and promote a healthier gut microbiota in adult cats. The findings support the potential of this strain to colonize the feline intestine, suggesting that further studies in larger populations and cats with gastrointestinal disorders are warranted [63].

Additionally, the impact of probiotic supplementation on fecal microbiota and characteristics in adult cats was investigated, while also assessing the effectiveness of different microencapsulation methods and storage stability. Eighteen cats were divided into three groups: a control group fed a standard commercial diet, a group receiving the same diet coated with probiotics, and a third group given a synbiotic diet containing freeze-dried probiotics and fructo-oligosaccharides (FOS). The probiotic blend included *L. acidophilus*, *L. casei*, *L. lactis*, *Bif. bifidum*, *E. faecium*, and *S. cerevisiae* encapsulated using either FOS or gum Arabic via freeze drying or spray drying. The results showed that freeze drying, particularly with FOS as the encapsulating agent, preserved microbial viability more effectively, achieving high bacterial counts of LAB and enterococci. A fecal analysis revealed a significant increase in LAB in the cats fed probiotic and synbiotic diets, indicating positive modulation of the gut microbiota. However, microbial viability declined over time during storage, highlighting the need for protective encapsulation strategies to safeguard probiotic function against environmental and processing stressors. These findings highlight the significance of formulation techniques in maintaining the stability and efficacy of probiotics in feline diets [64].

A recent study explored the potential of kefir as a natural probiotic supplement in Angora cats by evaluating its effects on gut microbiota, selected blood parameters, and fecal quality. Seven healthy cats were administered commercial kefir orally (30 mL/kg daily) for 14 days. The fecal and blood samples collected before and after supplementation revealed that kefir intake significantly increased beneficial gut microorganisms, including total mesophilic aerobic bacteria, *Lactococcus*, *Lactobacillus*, and yeast populations, while notably decreasing *Enterococcus* levels. Despite these microbial changes, no significant alterations were detected in most hematological and biochemical parameters, except for a reduction in lactate dehydrogenase activity and an increase in potassium levels. Additionally, kefir administration did not affect body weight, body condition score, fecal consistency, or fecal moisture. These findings suggest that daily kefir supplementation may enhance intestinal microbiota in cats without causing adverse health effects, supporting its potential role as a safe and functional dietary additive [65].

The impact of *L. reuteri* DSM 32264 supplementation on Persian cats has been reported. The results showed that administering DSM 32264 improved intestinal health by increasing beneficial *Lactobacillus* populations and reducing harmful bacteria, such as *E. coli*. Additionally, the probiotic contributed to better fecal quality, evidenced by firmer, well-formed stools and decreased fecal moisture, without affecting body weight or overall body condition. These findings suggest that DSM 32264 effectively supports gut microbial balance and promotes digestive wellness in healthy adult Persian cats [66].

A recent study investigated the effects of probiotic strains derived from feline feces (*L. plantarum* L-27-2 and *Pediococcus lactis* L-14-1) on lipid metabolism, inflammation, and gut microbiota in British shorthair cats. Both strains demonstrated the ability to lower blood triglycerides and low-density lipoprotein cholesterol levels while elevating high-density lipoprotein cholesterol levels. Additionally, *L. plantarum* L-27-2 significantly reduced the inflammatory marker, IL-6. An analysis of fecal microbiota revealed that these probiotics modulated specific bacterial genera associated with gut health, indicating their role in maintaining microbial balance. These results highlight the promising role of L-27-2 and L-14-1 as functional probiotics that can support metabolic and gastrointestinal health in cats. Further studies using advanced molecular techniques and longer-term trials are warranted to better understand their mechanisms and sustained benefits [67].

Furthermore, probiotics have shown promise in modulating oral microbiota and enhancing oral health in humans and rodents, yet their effects on feline oral microbiota remain largely unexplored. In a 42-day study, healthy cats supplemented with *Bif. animalis* subsp. *lactis* HN019, *L. acidophilus* NCFM, and *L. casei* LC-11 exhibited notable shifts in their oral microbial communities. An analysis of samples from the gingiva, tooth surfaces, and tongue revealed that the probiotic supplementation promoted beneficial bacterial genera, including *Moraxella*, *Actinomyces*, and *Frederiksenia* in the gingiva, as well as *Bergeyella* and *Streptococcus* on the teeth. Concurrently, there was a reduction in the abundance of potential pathogens such as *Bacteroides*, *Desulfovibrio*, and *Filifactor* in the gingiva, and *Porphyromonas* and *Treponema* on the tongue. These results suggest that probiotics may selectively enhance beneficial oral bacteria while suppressing harmful species, indicating their potential role in supporting oral health in cats. Further investigation is warranted to confirm its efficacy in preventing or managing feline oral diseases [68].

The impact of commercial *S. cerevisiae* products containing active metabolites, such as beta-glucans, nucleotides, organic acids, polyphenols, amino acids, vitamins, and minerals, on nutrient digestibility, fecal microbiota, fermentation products, and immune markers in healthy adult cats was evaluated. Twenty-seven cats were assigned to three groups: a control group and two treatment groups receiving the yeast additive at 0.3% and 0.6% inclusion levels. Supplementation improved the apparent digestibility of crude fiber and ash without altering food intake, fecal output, or fecal consistency. Notably, the 0.3% inclusion level significantly increased fecal lactic acid and reduced isovaleric acid, suggesting enhanced fermentative activity. A significant reduction in *C. perfringens* was also observed, indicating a favorable shift in gut microbiota. No significant effects were noted in fecal pH, ammonia levels, biogenic amines, short- or branched-chain fatty acids (except isovaleric acid), or immune function. These results suggest that *S. cerevisiae* supplementation at the tested doses exerts prebiotic effects that support gastrointestinal health without compromising nutrient digestibility. Further research is needed to explore the potential benefits of higher inclusion levels in feline diets [69]. Recently, the effect of *S. cerevisiae* var. *boulardii* (strain DSM 34246) on the intestinal health of adult Chartreux cats was evaluated. Fourteen healthy cats were randomly assigned to either a control group or a treatment group receiving the probiotic (5 × 10^9^ CFU/kg of feed) for 35 days. Various health parameters, including body condition score (BCS), fecal quality (assessed by fecal score, penetrometer measurements, dry matter, and moisture), and fecal IgA levels, were monitored. The probiotic supplementation led to significant improvements in BCS, fecal characteristics, and immune markers, indicating enhanced gut health without affecting body weight. These findings suggest that *S. boulardii* supplementation supports intestinal stability and overall physiological well-being in healthy cats. Further research is warranted to explore its therapeutic potential in cats with intestinal disorders [70].

Moreover, Veir et al. [71] examined the immunomodulatory potential of the *En. faecium* strain SF68 in kittens by assessing their immune responses to a multivalent vaccine. The kittens received a palatability enhancer either with or without *E. faecium* SF68 at a daily dose of 5 × 10^8^ CFU. The probiotic strain was recovered from the feces in most treated kittens, indicating successful gastrointestinal passage. While supplementation had no impact on growth or development, a significant increase in CD4+ lymphocyte levels was observed in the probiotic group. However, no significant differences were noted in other measured immune parameters. The study concluded that while *E. faecium* SF68 did not negatively impact development or immune function, it also had a limited influence on broader immune markers under the conditions tested. Future studies may benefit from incorporating challenge models, such as exposure to virulent feline herpesvirus 1, to assess the potential protective effects of probiotic supplementation [71].

The effects of probiotic supplementation (*B. amyloliquefaciens* SC06 and *B. subtilis* 10) on diarrhea prevention, gut microbiota composition, and metabolic profiles in healthy pet Ragdoll cats were investigated. Twenty healthy cats were assigned to either a control diet or a diet supplemented with 3 × 10^9^ CFU/kg of feed for 28 days. The results demonstrated that probiotic supplementation significantly reduced the incidence of soft stools and diarrhea. Immunologically, the treated cats showed decreased pro-inflammatory cytokines IL-1β and IL-6, alongside increased anti-inflammatory IL-10 levels. A microbiota analysis revealed an increased abundance of Phylum Patescibacteria and genus *Plectosphaerella*, while the populations of Phylam Firmicutes, genus *Gemmatimonadetes*, *Ruminococcaceae_UCG-005*, *Ascochytahe*, and *Saccharomyces* were reduced. Additionally, fecal SCFAs, particularly acetic and butyric acids, were elevated in the supplemented group. Metabolomic profiling revealed significant changes in serum metabolites, including elevated eugenitol and methyl sulfate, which correlated strongly with shifts in microbiota and immune modulation. Overall, these findings suggest that compound *Bacillus* supplementation can beneficially modulate gut microbiota, enhance metabolic health, and reduce diarrhea in pet cats [72].

The impact of *Lactobacillus* strain L11 on immune function, nutrient metabolism, and gut health in cats was investigated. Twelve healthy adult cats were divided into a control group and a treatment group receiving L11 supplementation over 28 days. The results showed a significant reduction in blood triglyceride levels in the cats treated with L11, indicating potential benefits in managing feline obesity. Additionally, a fecal analysis revealed a 30% increase in secretory IgA, suggesting an enhanced immune response, alongside an increase in *Bifidobacteria*. Importantly, the levels of harmful fecal compounds, including indole and 3-methylindole, were substantially decreased, reflecting improved intestinal health. These findings suggest that L11 has a positive influence on fat metabolism and modulates the gut microbiota, contributing to improved nutrient digestibility and reduced odor emissions. Overall, L11 presents as a promising probiotic candidate for supporting gut health and immune function in cats [73].

The impacts of supplementing the diet of healthy Chartreux cats with *B. clausii*, a probiotic known for its antimicrobial and immunomodulatory benefits, were studied. Over a 42-day double-blind trial involving 14 neutered cats, researchers monitored body weight, body condition, and fecal health parameters. The results showed that the cats receiving Bacillus clausii experienced significant improvements in body condition score and fecal quality, including increased fecal dry matter and decreased moisture content. These improvements suggest enhanced gut functionality without adverse effects on body weight. Given its resilience and proven human health applications, *B. clausii* appears to be a valuable probiotic for promoting gastrointestinal well-being in felines. Further investigation is recommended to assess its efficacy in cats with gastrointestinal disorders [74].

Immune deficiencies are a common concern in kittens, particularly during early developmental stages when their immune systems are still maturing [17]. The deficiencies can significantly compromise their health, increasing their vulnerability to infections and inflammatory conditions. Recent research has explored the use of dietary supplements, specifically those containing lactoferrin and the probiotic strain *L. plantarum*, as potential interventions to support immune and gastrointestinal health in kittens [17].

Evidence indicates that supplementation with lactoferrin and *L. plantarum* can significantly enhance immune responses. Notably, the levels of key immunoglobulins (IgA and IgG) were elevated by 14.9% and 14.2%, respectively. These increases suggest improved mucosal and systemic immunity, both of which are critical in protecting young animals from pathogens. The supplementation also led to a 28.7% increase in catalase activity, highlighting a reduction in oxidative stress and an improvement in antioxidant defense mechanisms. This antioxidative support is particularly important in growing kittens, as oxidative damage can impair the function of immune cells and overall development [17].

In terms of gastrointestinal (GI) health, the results were equally promising. The population of beneficial gut bacteria, especially *Lactobacillus*, increased dramatically from 4.13% to 79.03% over the study period. This shift in microbiota composition not only supports nutrient absorption and digestive efficiency but also contributes to immune regulation and resistance to enteric pathogens. Such microbial modulation is a crucial aspect of gut health, particularly in young animals whose microbiomes are still forming [17].

Additionally, the study [17] reported significant reductions in pro-inflammatory cytokines, with IL-2 levels decreasing by 13.94%, TNF-α by 26.46%, and IFN-γ by 19.45%. These changes reflect a more balanced immune response, reducing the likelihood of chronic inflammation and its associated health risks. Improvements were also observed in external indicators of well-being, such as coat quality and alertness, further suggesting systemic benefits beyond immune and GI health.

These findings collectively support the role of lactoferrin and *L. plantarum* as effective dietary components in improving the overall health status of kittens. Beyond the immediate physiological improvements, such supplementation strategies offer the potential to reduce the reliance on antibiotics, which is crucial given the growing concern over antimicrobial resistance. By enhancing natural immunity and supporting microbial balance, these interventions align with sustainable approaches to pet health management [17].

A recent pilot study evaluated the safety and potential gut health benefits of a novel supplement composed of 26 biotic components, including prebiotics, probiotics, and postbiotics, formulated based on evidence from feline and canine research. In a short-term trial involving three healthy cats, the supplement was well tolerated with no abnormalities observed in hematological or biochemical parameters. Notably, significant reductions in blood glucose and total cholesterol were recorded, alongside an increase in both T and B lymphocyte populations, suggesting immunomodulatory effects. Additionally, fecal pH significantly decreased, and two-thirds of the cats exhibited elevated levels of total organic acids, indicating improved fermentation activity. While no major shifts in fecal microbiota composition were detected at the genus level, a decrease in the Firmicutes/Bacteroidetes (F/B) ratio was observed in all cats receiving the supplement, suggesting a potential regulatory effect on the gut microbial balance. Despite the promising outcomes, the small sample size and short treatment duration highlight the need for further studies to confirm the long-term safety and efficacy in the feline population [75].

A recent study investigated the effects of yogurt enriched with postbiotics on feline gut health and immunity. Eighteen adult cats were divided into three groups: control, yogurt only, and yogurt with 2% postbiotics. After 21 days, the group receiving postbiotic-fortified yogurt showed significant reductions in serum levels of total bilirubin, bile acids, triglycerides, glucose, urea nitrogen, creatinine, and inflammatory markers (TNF-α and IL-6), while levels of secretory IgA were significantly increased. A microbiota analysis revealed a higher abundance of Bifidobacteria and enhanced microbial stability in the postbiotic yogurt group. Additionally, beneficial strains such as *Str. salivarius* subsp. *thermophilus* were successfully established. These findings indicate that postbiotic-enhanced yogurt may support immune function, reduce systemic inflammation, and promote a healthier gut microbiome in cats. Given its accessibility and nutritional value, this type of yogurt could serve as a promising dietary supplement in feline care, though further studies are needed to determine long-term safety and optimal dosing [76].

The impact of complex probiotic supplementation (*Bif. animalis* subsp. *lactis* BX-259, *Lpb. plantarum* LP-301, and *Lcb. rhamnosus* LR-78; 1:1:1 ratio) on gastrointestinal health in young kittens, a life stage marked by vulnerability to microbiome disturbances, was investigated. Following a brief acclimation period, the kittens receiving the probiotic formulation exhibited a significantly lower incidence of constipation compared with controls. Although the overall microbial diversity remained similar between the groups, a metagenomic analysis revealed an increased abundance of Bifidobacterium animalis and decreased levels of potentially harmful bacteria such as *Lachnospiraceae* bacterium 2_1_58FAA, *Lachnospiraceae* bacterium 1_1_57FAA, and *A. intestini* in the probiotic group. These findings suggest that early probiotic intervention can support gut health in kittens by modulating intestinal flora and reducing inflammation-related microbial populations, making it a promising nutritional strategy for promoting healthy development in the early life stages [77].

The juvenile phase in cats is a vital period for the development of a healthy and functional intestinal microbiome. A recent study used metabolomics to assess the effects of a complex probiotic supplement on gut health in kittens. Over 14 days, the kittens receiving the probiotic formulation showed significant shifts in specific intestinal metabolites linked to inflammation and gastrointestinal function. Notably, the levels of beneficial compounds such as vitamin K3, methylmalonyl carnitine, and lysyl hydroxyproline increased, while pro-inflammatory and constipation-associated metabolites, including gamma-glutamyl-L-putrescine, myristic acid, and glycodeoxycholic acid, were reduced. These changes suggest that complex probiotics may support intestinal health in kittens by modulating the metabolic pathways involved in inflammation and bowel regulation. The findings provide a strong foundation for the development of targeted probiotic supplements aimed at enhancing gastrointestinal function during early feline development [78].

## 5. Health Benefits of Probiotic Supplementation in Cats with Health Issues

Probiotic supplementation has also been explored for its therapeutic potential in cats with diseases, targeting conditions such as chronic diarrhea, obesity, asthma, and treatment-resistant enteropathies (Table 2).

In addition, *B. licheniformis*-fermented products (BLFPs) have demonstrated potential probiotic benefits, including antibacterial and anti-inflammatory effects. BLFP supplementation in cats with chronic diarrhea resulted in clinical improvement in most subjects within one week. The treatment was associated with favorable shifts in gut microbiota, including reduced levels of *C. perfringens* and an increased abundance of beneficial bacterial taxa within the *Clostridium* cluster XIVa, such as *Blautia* spp., *R. torques*, and *R. gnavus*. Although some diarrheal cats exhibited persistent microbial imbalances, such as an elevated Firmicutes/Bacteroidetes ratio, the overall findings support the potential role of BLFPs in alleviating gastrointestinal symptoms and modulating intestinal microbial communities in affected felines. Additional research is necessary to fully elucidate the functional implications of these microbial changes on gut homeostasis [6].

A controlled, double-blind study evaluated the effect of *E. faecium* SF68 supplementation on the gastrointestinal health of shelter animals. The experimental cats were supplemented with probiotics for 8 weeks. The cats receiving the probiotic experienced a significantly lower incidence of diarrhea lasting two or more days compared with the placebo group, suggesting a beneficial role of the probiotic in promoting gut health. The probiotic’s effect is likely due to its ability to inhibit gastrointestinal colonization by pathogens rather than through systemic immune modulation, as the treatment duration per animal was brief. These findings suggest that the use of probiotics in shelters could improve animal welfare by reducing diarrhea-related issues; however, a cost–benefit analysis would be necessary to assess the feasibility of implementation on a larger scale [79].

Similarly, the effects of *E. faecium* SF68 supplementation in overweight and obese, yet otherwise healthy, cats were reported to have no significant impact on food consumption, body weight, body composition, or metabolic indicators. The cats were fed ad libitum and monitored for intake, weight, and various blood parameters, including glucose, lipids, insulin, leptin, and adiponectin. No measurable differences were observed between the probiotic-treated and control groups. These results suggest that short-term probiotic administration alone may not influence the metabolic outcomes in obese feline populations. However, further research with extended treatment durations, varied probiotic strains, or the inclusion of calorie restriction is recommended to better understand the potential benefits [80].

Moreover, a recent placebo-controlled clinical trial investigated the effects of oral multi-strain probiotics in asthmatic cats receiving anti-inflammatory glucocorticoids. The study, involving 13 client-owned cats, aimed to evaluate improvements in respiratory symptoms, immune modulation, and microbial diversity across respiratory, blood, and oropharyngeal sites. The results showed no significant differences between the probiotic and placebo groups in terms of clinical signs, airway eosinophil counts, microbial diversity, or the expression of key immune markers and cytokines. These findings suggest that short-term oral probiotic supplementation does not provide measurable clinical or immunological benefits in feline asthma under the conditions tested. Future research may explore alternative probiotic formulations, delivery methods, or longer treatment durations to determine potential benefits [81].

Finally, a retrospective case series evaluated the use of commercially available oral fecal microbiota transplantation (FMT) capsules as an adjunct therapy for chronic enteropathies in cats that were unresponsive to standard treatments. Involving five dogs and three cats, the study reported significant clinical improvements after one month of daily FMT capsule administration, as measured by standardized disease activity indices (Feline Chronic Enteropathy Activity Index). Key symptoms, including diarrhea, vomiting, anorexia, and lethargy, showed marked resolution without any observed adverse effects. These findings suggest that oral FMT capsules may offer a promising, well-tolerated option for managing chronic gastrointestinal conditions in companion animals [82].

Although outcomes can vary based on the specific condition and probiotic strain used, multiple studies have reported promising clinical improvements, particularly in managing gastrointestinal disorders. Overall, current research highlights the beneficial effects of probiotics on feline health, demonstrating improvements in gut microbiota balance, fecal quality, immune response, and metabolic function, with no adverse side effects. These results support the use of probiotics as functional dietary tools to maintain and enhance the well-being of domestic cats. Continued investigation, especially in cats with clinical illnesses or compromised immune systems, is crucial for identifying optimal strains, dosages, and treatment durations, as well as harnessing the full therapeutic potential of probiotics in veterinary medicine.

Current research indicates that while probiotics show potential in supporting feline gastrointestinal health, they are not yet clinically viable replacements for antibiotics in treating infectious diseases. A recent meta-analysis evaluating biotic supplementation in cats reported the beneficial effects of probiotics, particularly on fecal consistency in five out of seven trials, but the overall certainty of evidence was low and inconsistent across studies [1]. Similarly, another study suggested that complex probiotics significantly modified intestinal metabolites and reduced inflammation in kittens, suggesting a potential role in preventive gut health management [78]. However, these effects are largely limited to gastrointestinal outcomes and have not yet been extended to systemic infectious disease contexts. While not feline-specific, studies into quorum sensing inhibitors have shown that certain non-antibiotic agents can enhance the efficacy of antibiotics and reduce bacterial virulence through synergistic mechanisms, offering a parallel model for adjunctive therapies [83]. Overall, probiotics may help reduce the need for antibiotics in mild or non-serious cases, supporting better antibiotic use. However, existing scientific evidence does not support probiotics as a full replacement for antibiotics when treating infectious diseases in cats.

## 6. Mechanisms of Action of Probiotics

**Figure 1:**
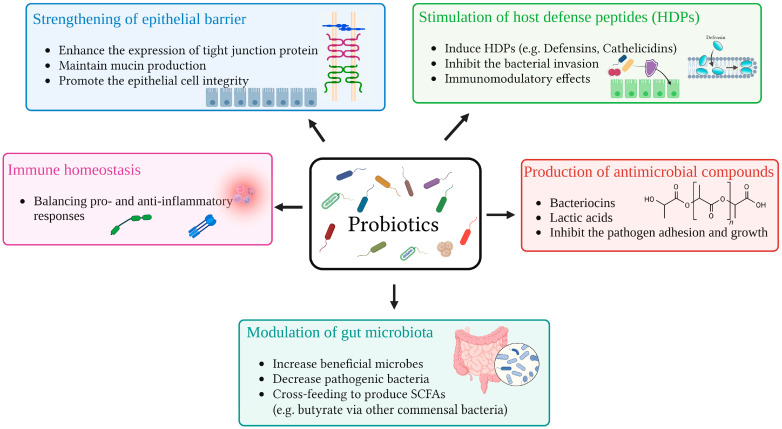
Possible mechanisms underlying the health benefits of probiotics in cats: This diagram illustrates the core mechanisms by which probiotics, particularly strains of *Lactobacillus* and *Bifidobacterium*, support gastrointestinal health in cats. These include modulation of the gut microbiota through promotion of beneficial microbial populations, suppression of pathogenic organisms, and stimulation of SCFAs production via cross-feeding interactions. Probiotics also enhance the integrity of the intestinal epithelial barrier by upregulating tight junction proteins and sustaining mucin secretion. Additionally, they stimulate the host’s production of antimicrobial peptides, which contribute to both pathogen defense and immune regulation. Furthermore, probiotics synthesize antimicrobial substances such as bacteriocins and lactic acid that inhibit pathogen colonization. Collectively, these effects promote immune homeostasis by regulating both pro-inflammatory and anti-inflammatory pathways.

Probiotics exert a wide range of beneficial effects on the host through multiple interconnected mechanisms that enhance gut and immune health [84,85]. Probiotics are known to exert their positive effects on host health through various mechanisms (Figure 1), including interactions with the native gut microbiota and communication with host cells. These beneficial microbes contribute to gut health by promoting a balanced microbial environment, reinforcing the epithelial barrier, and supporting immune system regulation either directly or indirectly [16,86,87,88].

One of the primary ways probiotics prevent pathogenic invasion is through colonization resistance and the secretion of antimicrobial substances, such as bacteriocins and organic acids like lactic acid [89]. Although species from the *Lactobacillus* and *Bifidobacterium* genera do not produce butyrate themselves, they can facilitate the activity of other gut microbes that do, thereby enhancing the production of SCFAs, which play diverse and vital physiological roles [90,91]. Additionally, probiotics can promote health by stimulating the production of host defense peptides (HDPs). Specific strains including *L. casei*, *L. paracasei*, *L. plantarum*, *B. breve*, *A. muciniphila*, *Ba. thetaiotaomicron*, and *E. coli* Nissle 1917 have been shown to induce HDP production in both humans and animals [92,93,94,95]. HDPs, also referred to as antimicrobial peptides, are small, positively charged, and amphipathic molecules that are widely expressed in epithelial tissues and immune cells such as phagocytes [96]. With roles in both antimicrobial defense and immune regulation, HDPs are considered a crucial part of the host’s immune response [96,97].

Probiotic supplementation improved feline health status by targeting the gut microbiome, immune system, oxidative stress, and intestinal barrier integrity as observed in other hosts. One of the primary mechanisms involves modulating the gut microbiota. *Bif. animalis* subsp. *lactis* CECT 8145 supplementation for 90 days significantly enhanced microbial diversity in the feline gut, increasing the relative abundance of beneficial bacteria. It has been observed that CECT 8145 supplementation suppresses the pathogenic pathways, including biofilm formation, membrane transport, and bacterial motility. In contrast, probiotics have been shown to improve carbon metabolism pathways, amino acid biosynthesis, and mucosal immune function [98]. With the supplementation of live and heat-killed *Bif. animalis*, treated cats demonstrated marked reductions in inflammatory cytokines, along with elevated levels of beneficial short-chain fatty acids linked to colonic health [99].

The supplementation of *Bif. lactis* and *L. plantarum* effectively improved feline health. The probiotics enhanced immune responses by increasing serum IgA and cytokine levels. The host antioxidant capacity was enhanced, leading to a reduction in oxidative stress. The study found that probiotic supplementation improved the integrity of the gut barrier, as shown by decreased plasma levels of diamine oxidase and D-lactate, indicators of reduced intestinal permeability. A fecal microbiota analysis revealed positive changes in gut microbial composition, indicating an improvement in gut health. The study claimed that probiotic supplementation can strengthen immune function, antioxidant system, and gastrointestinal integrity in cats [100].

These findings highlight the therapeutic potential of probiotics in promoting both gut and overall health in cats; however, further studies are necessary to elucidate the precise mechanisms.

## 7. Limitations and Future Perspectives

While the current review highlights the potential benefits of probiotic supplementation for the health and well-being of cats, several limitations are consistently reported across studies. A major recurring issue is the short duration of intervention periods, with many studies lasting only a few weeks. Longer-term investigations are essential to evaluate the sustained effects of probiotic administration, particularly on chronic conditions such as oral and gastrointestinal diseases [68,81].

Another common limitation is the small sample size used in many experimental designs, often with fewer than 10 experimental cats per group. This restricts the statistical power and the generalizability of findings. Increasing the number of subjects in future studies would not only strengthen conclusions but also enable more sophisticated analyses, such as metabolomics and multi-omics approaches, offering deeper insight into the mechanisms of probiotic action [67,81].

The lack of breed diversity and the frequent use of only healthy cats further limit the applicability of results to broader feline populations. Future studies should include a more heterogeneous sample, incorporating cats of different breeds, ages, and health conditions, including those with existing oral or gastrointestinal diseases [68,70]. Moreover, studies involving client-owned animals introduce real-world variability but also pose challenges such as inconsistent environments, diets, and treatment compliance, all of which can influence study outcomes [81].

Another gap in the literature is the limited assessment of immune and inflammatory biomarkers. Few studies have evaluated parameters such as fecal calprotectin, blood cell profiles, cytokine levels, or acute phase proteins. Including these markers could enhance the understanding of how probiotics influence systemic and mucosal immune responses [70].

In addition, while changes in the gut and oral microbiota are commonly reported, the functional implications of these shifts remain poorly understood. Further research is needed to clarify the relationship between microbial community composition and host health outcomes, particularly in the context of maintaining gut and oral homeostasis [20,82].

Finally, studies using fecal microbiota transplantation (FMT) or multi-strain probiotics would benefit from standardized protocols and longitudinal microbiome analyses to better elucidate their mechanisms and therapeutic potential [81,82]. In conclusion, current evidence shows that probiotics can benefit feline health. However, future studies should include longer treatment periods, more diverse and larger groups of cats, the detailed analysis of health markers, and a better understanding of how probiotics work to unlock their potential in veterinary care.

## 8. Conclusions

The literature shows the promising role of probiotics in promoting the health and well-being of domestic cats, particularly through their capacity to modulate gut microbiota, support immune function, and improve clinical outcomes in both healthy and diseased populations. Probiotic strains such as *Lactobacillus* spp., *Bifidobacterium* spp., *Bacillus* spp., *E. faecium*, and *S. boulardii* have demonstrated beneficial effects on gastrointestinal function, microbial balance, metabolic regulation, and immune responsiveness, with minimal adverse effects reported. These findings support the integration of probiotics as functional dietary tools in feline health management, offering a safer and more sustainable alternative to conventional antimicrobial treatments. However, the current body of research has several limitations. Future investigations are necessary to elucidate the strain-specific mechanisms and optimize clinical applications. In conclusion, probiotics hold significant potential to enhance preventive care, support disease management, and improve the overall quality of life in domestic cats.

## Figures and Tables

**Table 1:** Effects of probiotic supplementation on clinical and physiological health markers in domestic cats.

Probiotics	Cats’ Details	Dose and Duration	Observed Outcomes	References
*L. acidophilus* DSM13241	Sex: Not mentioned. Age: 4–5.5 years. Breed: Domestic shorthair cats.	2 × 10^8^ CFU/day for 4.5 weeks.	Altered the balance of gut microflora in healthy cats and provided systemic immunomodulatory benefits.	[59]
Proviable^®^-DC (Nutramax Laboratories Veterinary Sciences, Inc., Lancaster, SC, USA) [*E. faecium* (NCIMB 30183), *Str. salivarius* ssp. *thermophilus* (NCIMB 30189), *Bif. longum* (NCIMB 30179), *L. acidophilus* (NCIMB 30184), *L. rhamnosus* (NCIMB 30188), *L. plantarum* (NCIMB 30187), *L. delbrueckii* ssp. *bulgaricus* (NCIMB 30186)]	Sex: Not mentioned. Age: 0.7–6.7 years. Breed: Domestic shorthairs, domestic long hair, domestic medium hair, Persian, and mixed cats.	5 × 10^9^ CFU per capsule; one capsule per day for 21 days.	Increased the probiotic bacteria in feces without changing dominant bacterial phyla or causing adverse effects.	[60]
1% Galacto-oligosaccharides and *Bif. pseudocatenulatum* (BP-B82)	Sex: Not mentioned. Age: 1–6 years. Breed: European domestic shorthair cats.	10^10^ CFU/day for 15 days.	Improved the intestinal microbiota.	[61]
Proviable^®^-DC synbiotic capsule	Sex: Castrated males (*n* = 9), spayed females (*n* = 7). Age: 7–10 years. Breed: Domestic shorthair cats.	75 mg clindamycin with feed per day; after 1 h, 2 capsules of 5 × 10^9^ CFU for 3 weeks.	Administering a synbiotic after clindamycin reduced hyporexia and vomiting, with benefits lasting at least six weeks, though it did not decrease antibiotic-associated diarrhea.	[62]
*L. acidophilus* D2/CSL (CECT 4529)	Sex: Male (*n* = 3), female (*n* = 7). Age: >9 years. Breed: Maine Coon cats.	5 × 10^9^ CFU/kg feed per day for five weeks.	Improved fecal quality and gut health in healthy adult cats.	[63]
*L. acidophilus*, *L. casei*, *L. lactis*, *Bif. bifidum*, *E. faecium*, and *S. cerevisiae*	Sex: Male (*n* = 9), female (*n* = 9). Age: 3 ± 0.84 years. Breed: Not mentioned.	10^9^ CFU of each strain per gram. 1 or 2 g/cat per day for 20 days.	Improved the fecal microbiota of cats by increasing the lactic acid bacteria counts.	[64]
Commercial kefir	Sex: Male (*n* = 5), female (*n* = 2). Age: 3.3 ± 2.5 years. Breed: Angora cats.	30 mL/kg of body weight for 14 days.	Enhanced intestinal microbiota diversity in Angora cats.	[65]
*L. reuteri* NBF 2 DSM 32264	Sex: Male (*n* = 4), female (*n* = 8). Age: >1 year. Breed: Persian cats.	5 × 10^9^ CFU/kg of feed for 35 days.	Improved fecal quality in Persian cats. Increased Lactobacilli concentration and reduced coliforms.	[66]
*L. plantarum* L-27-2 or *P. lactis* L-14-1	Sex: Male (*n* = 4), female (*n* = 8). Age: 2–5 years. Breed: British shorthair cats.	1 × 10^9^ CFU of each strain/kg of body weight/day for 28 days.	Improved gut health and obesity management.	[67]
*Bif. animalis* subsp. *lactis* HN019, *L. acidophilus* NCFM, and *L. casei* LC-11	Sex: Male (*n* = 6), female (*n* = 6). Age: 2–4 years. Breed: Chinese domestic cats, British shorthair cats.	1 × 10^10^ CFU/kg feed per day for 42 days.	Improved the feline oral microbiota by promoting beneficial bacteria and inhibiting pathogens.	[68]
*S. cerevisiae* (YAM)	Sex: Not mentioned. Age: 9.44 ± 5.35 years. Breed: Not mentioned.	0.3 or 0.6% YAM in controlled diet. 3 times per day for 37 days.	The additive acts as a prebiotic by altering fecal fermentation and microbiota, without affecting the digestion of protein or dry matter.	[69]
*S. cerevisiae* DSM 34246 (Canobios-BL) var. *boulardii*	Sex: Male (*n* = 4), female (*n* = 10). Age: 3–6 years. Breed: Chartreux cats.	5 × 10^9^ CFU/kg feed per day for 35 days.	Supports gut health and maintains physiological well-being in breeding cats.	[70]
*E. faecium* strain SF68 (NCIMB10415)	Sex: Not mentioned. Age: 6 weeks. Breed: Not mentioned.	5 × 10^8^ CFU/day.	Increased the CD4+ lymphocyte percentages.	[71]
*B. amyloliquefaciens* SC06 (BaSC06) and *B. subtilis* 10 (B10)	Sex: Not mentioned. Age: 1–2 years. Breed: Ragdoll cats.	3 × 10^9^ CFU/kg for 28 days.	Increased the levels of eugenitol and methyl sulfate in the serum. Increased the total SCFAs, acetic acid, and butyric acid in the stool. Lowered IL-1β and IL-6 levels in the serum.	[72]
*L. plantarum*	Sex: Female (*n* = 12) Age: about 2 years. Breed: Not mentioned.	1 × 10^9^ CFU/kg feed per day for 28 days.	Enhanced gut health and immune function in cats, potentially linked to lipid metabolism.	[73]
*B. clausii*	Sex: Neutered males (*n* = 14). Age: 6.2 ± 2.8 years. Breed: Chartreux cats.	1 × 10^6^ CFU/day for 42 days.	Improved gut health and overall well-being in cats.	[74]
Mixture of inactivated Bifidobacteria and lactic acid bacteria	Sex: Male (*n* = 3) Age: Average 5.33 years. Breed: Domestic shorthair cats.	4.5 g per day.	Improved gut health and microbiota.	[75]
Inactivated *P. lactis*	Sex: Male (*n* = 9); female (*n* = 9). Age: Approximately 3 years. Breed: Ragdoll, Russian Blue, and British shorthair intact cats.	Yogurt + 2% postbiotics, 50 g per day for 21 days.	Improved gut health and immunity.	[76]
*Bif. animalis* subsp. *lactis* BX-259, *Lpb. plantarum* LP-301, and *Lcb. rhamnosus* LR-78 (1:1:1 ratio)	Sex: Male (*n* = 8); female (*n* = 16). Age: 3–4 months. Breed: Not mentioned.	1.5 × 10^9^ CFU/day for 14 days.	Improved intestinal microbiota and constipation and promoted intestinal health. Regulated the intestinal metabolites and reduced intestinal inflammation.	[77,78]

**Table 2:** Impact of probiotic supplementation on clinical health parameters in cats with underlying health conditions.

Probiotics	Cat’s Details	Dose and Duration	Health Issue	Observed Outcomes	References
*B. licheniformis*-fermented products (BLFPs)	Sex: Castrated males (7); spayed females (5). Age: 3–15 years. Breed: Domestic shorthair, American shorthair, and Scottish fold.	1.1 mg/kg BLFP in a capsule. One capsule per day for 7 days.	Chronic diarrhea	BLFPs reduced *Clostridium perfringens* levels and improved gastrointestinal symptoms and fecal microbiota.	[6]
*E. faecium* SF68	Sex: Male (*n* = 57); female (*n* = 73); not recorded (*n* = 87). Age: Not mentioned. Breed: Stray (*n* = 143); feral (*n* = 74).	2.1 × 10^9^ CFU/g. 1 g per day for 4 weeks, followed by a 1-week washout period, then supplemented with placebo, and vice versa.	Diarrhea	Cats supplemented with SF68 experienced fewer episodes of prolonged diarrhea compared with the control group and showed improved gastrointestinal health.	[79]
*E. faecium* SF68	Sex: Probiotic group: castrated males (*n* = 6), female (*n* = 4). Control group: castrated males (*n* = 2), female (*n* = 5). Age: Probiotic group (mean of 8.5 years). Control group (mean of 9.8 years). Breed: Not mentioned.	5 × 10^8^ CFU/g in 10 g of feed/day for 8 weeks.	Overweight and obesity	No major effect on appetite, weight, body fat, or metabolism in healthy, overweight cats.	[80]
Multi-strain probiotics (Visbiome^®^ Vet capsules)	Sex: Castrated males (*n* = 10), spayed females (*n* = 3). Age: 2–13 years. Breed: Domestic shorthairs, mixed breed, Bobtail, and Siamese.	112.5 × 10^9^ cells per 0.45 g per capsule per day for 2 weeks.	Asthma	No significant improvement in respiratory symptoms, immune response, airway eosinophilia, and microbial composition.	[81]
Oral capsule fecal microbiota transplant (Kittybiome^®^)	Sex: Castrated male (*n* = 1), spayed females (*n* = 2). Age: 3–6 years. Breed: Ragdoll, Sphinx, and Exotic shorthair cat.	One month.	Chronic enteropathy	Demonstrated potential as a treatment for chronic enteropathies in cats that do not respond to standard therapies.	[82]

## Data Availability

No new data were created.

## Abbreviations

The following abbreviations are used in this manuscript:

USD	United States Dollar
SCFAs	Short-chain fatty acids
*B.*	*Bacillus*
*L.*	*Lactobacillus*
*Lpb.*	*Lactiplantibacillus*
*Lcb.*	*Lacticaseibacillus*
*Str.*	*Streptococcus*
*E.*	*Enterococcus*
*Es.*	*Escherichia*
*Bif.*	*Bifidobacterium*
*S.*	*Saccharomyces*
*P.*	*Pediococcus*
*R.*	*Ruminococcus*
*C.*	*Clostridium*
*A.*	*Acidaminococcus*
*Ba.*	*Bacteroides*
IBD	Inflammatory bowel disease
ALT	Alanine aminotransferase
N:L	Neutrophil-to-lymphocyte
WBC	White blood cells
CFU	Colony-forming unit
GOS	Galacto-oligosaccharides
AAGSs	Antibiotic-associated gastrointestinal signs
FOS	Fructo-oligosaccharides
LAB	Lactic acid bacteria
BCS	Body condition score
Ig	Immunoglobulin
DNC	Dietary nutritional combination
IgG	Immunoglobulin G
IgA	Immunoglobulin A
IgM	Immunoglobulin M
Control group	CON
GI	Gastrointestinal
TNF-α	Tumor necrosis factor-alpha
IFN-γ	Interferon gamma
IL-2	Interleukin-2
BLFP	*B. licheniformis*-fermented products
FMT	Fecal microbiota transplantation
HDPs	Host defense peptides
